# Comparison Between Angiotensin-Converting Enzyme Inhibitors and Angiotensin Receptor Blockers for Incidence of Lung Cancer: A Retrospective Study

**DOI:** 10.7759/cureus.14788

**Published:** 2021-05-01

**Authors:** Pardeep Kumar, Vinod Kumar, Manish Murlidhar, Aliya Fatima, Maha Jahangir, Dua Khalid, Muhammad Khizar Memon, Sidra Memon, Besham Kumar

**Affiliations:** 1 Medicine, Jinnah Sindh Medical University, Karachi, PAK; 2 Internal Medicine, Cleveland Clinic Abu Dhabi, Abu Dhabi, ARE; 3 Internal Medicine, Chandka Medical College Hospital, Larkana, PAK; 4 Internal Medicine, Jinnah Postgraduate Medical Centre, Karachi, PAK; 5 Anesthesiology, Civil Hospital Karachi, Karachi, PAK; 6 Internal Medicine, Jinnah Sindh Medical University, Karachi, PAK; 7 Internal Medicine, Liaquat University of Medical and Health Sciences, Hyderabad, PAK

**Keywords:** renin angiotensin alderosterone system, lung cancer, association, ace (angiotensin converting enzyme), angiotensin receptor blockers

## Abstract

Introduction: Angiotensin-converting enzyme inhibitor (ACEI) and angiotensin II receptor blockers (ARBs) are taken as the first treatment option for hypertensive patients. The various global trials have suggested that ACEIs and ARBs may increase risk of lung cancer; however, the results are contradictory and there is no local study available. This study is conducted to compare the incidence of lung cancers in patients on ACEIs and ARBs.

Methods: This retrospective study, conducted in a major cardiology unit of a tertiary care hospital in Pakistan, included patients diagnosed with hypertension, between 2005 and 2010, who were prescribed either ACEIs or ARBs. During the period of 2005 to 2010, 47,823 naïve hypertensive patients were reported in the outpatient department of the cardiology unit. Of which, 22,241 were prescribed ACEI and 25,582 were prescribed ARBs. After sorting patient data based on our inclusion criteria, n = 14,891 participants were included in the ACEI group and n = 19,112 participants were included in the ARB group.

Results: The incidence of lung cancer in the ACEI and ARB group was n = 165 and n = 160, respectively. In this study, the overall incidence rates of lung cancer in the ACEI and ARB cohorts were 12.2 and 16.6 per 10,000 person-years, respectively. The hazard ratio was 1.32 (95% confidence interval: 1.06-1.64; p-value: 0.01).

Conclusion: In this study, the incidence of lung cancer was relatively more among people using ACEIs than ARBs. Hence, patients undergoing long-term treatment with ACEIs need regular follow-up and proper scanning to avoid grave complications.

## Introduction

Angiotensin-converting enzyme inhibitors (ACEIs) and angiotensin II receptor blockers (ARBs) are drugs that block the body’s renin-angiotensin-aldosterone system (RAS) and are usually taken as the first treatment option for hypertensive patients [1,2]. These drugs can also be used as a secondary treatment for heart failure, post-myocardial infarction and chronic kidney diseases [3]. Despite their common usage, some unfavorable effects of these medications such as anaphylactic reaction, angioedema, renal failure, jaundice and hepatitis, and hyperkalemia-induced arrhythmia cannot go unnoticed. Moreover, ACEIs are known to be associated with benign cough production. ACEI is also seen to cause higher lung cancer risk compared with ARB [4].

A recent study has demonstrated that ACEIs are known to increase the lung cancer risk by 14%, compared with ARBs, which increased to 31% when ACEIs was used for 5 to 10 years. This is justified by the fact that it causes the accumulation of bradykinin and substance P (SP) in the alveoli of the lungs, which might potentially cause lung cancer [5]. Another research has also proven the fact that patients treated with ACEI were at an increased risk of lung cancer by 1.36 folds, whereas patients on ARB were at risk of 0.62 folds. This point towards the fact that patients who use ARB are less likely to get lung cancer compared with non-ARB users. [6]. However, data comparing ACEI and ARB for causing lung cancer are very limited from developing countries where ACEIs are frequently used to be a cheaper option; therefore, it is important to conduct a study to assess the incidence of lung cancer caused by these antihypertensive drugs.

## Materials and methods

This retrospective study was conducted in a cardiology unit of three tertiary care hospital in Pakistan in March 2021. Data of patients diagnosed with hypertension between 2005 and 2010, who were prescribed either ACEIs or ARBs, were included in the study. Patients with less than five years of follow-up data were excluded from the study. Similarly, patients who switched medicine, from ACEI either to ARBs or vice versa were also excluded from the study. Permission to retrieve data of patients was taken from the ethical review board of the institute.

**Figure 1:**
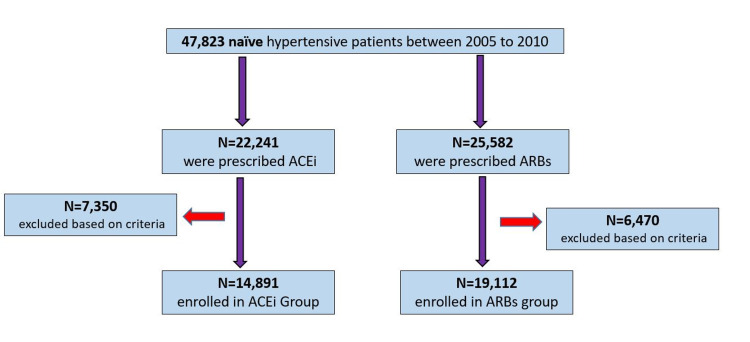
Selection of Participants ACEI; Angiotensin-converting enzyme inhibitor. ARBs; Angiotensin receptor blockers

During the period of 2005 to 2010, 47,823 naïve hypertensive patients were reported in the outpatient department of the cardiology unit. Of which, 22,241 were prescribed ACEI and 25,582 were prescribed ARBs. After sorting patient data based on our inclusion criteria, n = 14,891 participants were included in the ACEI group and n = 19,112 participants were included in the ARB group (Figure 1).

Age, gender, comorbidities, concomitant medication for hypertension and incidence of lung cancer was noted in the self-structured questionnaire. Statistical package for Social Sciences® software version 23.0 (SPSS; IBM Corp., Armonk, NY, USA) was used for data analysis. For numerical variables, data were expressed as mean ± standard deviation. Frequencies and percentages were used for categorical variables. ACEI and ARB group’s distribution of demographics, comorbidities and concomitant medication were compared. Differences were examined using Student’s t-test for continuous variables and the chi-square test for categorical variables. We measured the survival probability in the ACEI and ARB cohorts using the Kaplan-Meier method. A p-value of less than 0.05 meant that there is a significant difference between the two groups and the null hypothesis is not valid.

## Results

The mean age at the time of diagnosis of hypertension was in ACEI group was 49 ± 11 years and 50 ± 13 years in the ARB group. Comparison of further demographics is given in Table 1.

**Table 1:** Comparison of characteristics of patients undergoing treatment with ACEI or ARB ACEI: Angiotensin-converting enzyme inhibitor, ARB: Angiotensin receptor blocker, CLD: Chronic liver disease, CKD: Chronic kidney disease, DM: Diabetes mellitus, NS: non-significant, MI: Myocardial Infarction

Demographics of participants	ACEI group (n=14,891)	ARB group (n=19,112)	P-value
Age at the time of diagnosis of hypertension (Mean ± Standard Deviation)	49 ± 11	50 ± 12	NS
Male	7,854 (52.74%)	10,781 (56.41%)	NS
Smokers	5,021 (33.7%)	6,812 (35.65%)	NS
Comorbidity			
DM	7,652 (51.39%)	10,856 (56.80%)	NS
CLD	1,101 (7.39%)	1,651 (8.64%)	NS
CKD	2,022 (13.58%)	2,401 (12.56%)	NS
Hyperlipidemia	6,078 (40.82%)	7,998 (41.85%)	NS
Asthma	782 (5.25%)	1,021 (5.34%)	NS
Stroke	515 (3.46%)	600 (3.14%)	NS
MI	1,254 (8.42%)	1,601 (8.38%)	NS
Concomitant Medications for Hypertension			
Alpha blockers	131 (8.81%)	1,721 (9.00%)	NS
Beta blockers	6,052 (40.64%)	8,502 (44.49%)	NS
Calcium channel blockers	10,112 (67.91%)	13,281 (69.49%)	NS
Diuretics	6,565 (44.09%)	8,215 (42.98%)	NS

The mean follow-up time was 7.01 ± 3.02 years and 7.12 ± 3.35 years in the ACEI and ARB group, respectively. The incidence of lung cancer in the ACEI and ARB group was n = 165 and n = 160, respectively. In this study, the overall incidence rate of lung cancer in the ACEI and ARB cohorts were 12.2 and 16.6 per 10,000 person-years, respectively. The hazard ratio was 1.32 (95% confidence interval: 1.06-1.64; p-value: 0.01) (Table 2).

**Table 2:** Comparison of follow-up and incidence of lung cancer in both groups

	ACEI group (n= 14,891)	ARB group (n= 19,112)
Follow up time in years (Mean ± SD)	7.01 ± 3.02	7.12 ± 3.35
Person years (No. of participants x Mean follow up time)	104,385	136,077
Events (incidence of lung cancer)	165	160
Rate (event per 10,000 person-year)	15.86	11.7
Hazard ratio	1.32 (p-value: 0.01)	1

In Kaplan-Meier survival analysis, the survival probability of not having an event (lung cancer) was significantly higher in the ARB cohort than in the ACEI cohort (log-rank test, p-value = 0.002). In both ACEI and ARB groups, increased exposure to ARBs and ACEIs was associated with an incidence of lung cancer. However, there was no difference between both groups (Table 3).

**Table 3:** Comparison of incidence of development of lung cancer based on usage of medications over the number of years

No. of years on medication	ACEI group (Events) (n=165)	Intra group p-value	ARB group (Events) (n=160)	Intragroup p-value	Intergroup p-value
0-2	2 (1.2%)	< 0.00001	1 (0.6%)	< 0.00001	0.9
2-4	11(6.6%)		9 (5.6%)		
4-6	21 (12.7%)		19 (11.8%)		
6-8	28 (16.9%)		27 (16.8%)		
8-10	45 (27.2%)		50 (31.2%)		
10-12	65 (39.3%)		54 (33.7%)		

## Discussion

Our study demonstrates that the ACEI group is significantly more prone to getting lung cancer as compared to the ARB group. Kasper et al. in their study suggest that high cumulative ACEI doses may result in a modest increase in odds of lung cancer. However, they suggested lower dose showed no association [7].

On the contrary, two meta-analyses suggested no link between comparatively increased cancer risk and ACEIs because the events were found to be similar in both ARB and ACEI treated group and control group [8,9]. Bangalore et al. stated that patients treated with ACEI and ARB combination therapy were at a higher risk of cancer than those who were treated with only ACEI [8]. Conversely, the other proved that there was no increased cancer risk in patients who were treated with combination therapy and those with ACEI alone [9]. In these meta-analyses, the risk of cancer was associated more with combination therapy in three trials with longer follow-up time (mean = 48 months) and showed more link with individual treatment in four trials with shorter follow-up time (mean = 32 months) [8,9]. This suggests that confirmed findings to assess the outcomes of ARB and ACEI treatment would be obtained with a longer follow-up time. Another cohort study conducted in 2018, with a mean follow-up time of 6.4 years proved that ACEI had a stronger connection with causing lung cancer compared with ARB, which is consistent with the findings of our study [5].

The biological association between ACEIs and lung cancer has been explained by studies. Angiotensin-converting enzymes also metabolize bradykinin, a vasoactive substance known to be responsible for dilation [10]. Therefore, ACEI usage would lead to the accumulation of bradykinin in the lungs [11]. Bradykinin receptors have their sites on several cancerous tissues including lung cancer, and bradykinin can directly trigger the growth of lung cancer [11,12]. Bradykinin causes the release of vascular endothelial growth factor, which in turn would stimulate angiogenesis [13,14], and also indirectly affects lung cancer as it acts as a promoter of permeability of the vessels, by activating matrix metalloproteinase, which in turn leads to tumor invasion and metastases [14]. Additionally, ACEI causes the build-up of SP, which is usually observed in lung cancer and is known to be linked with angiogenesis and tumor proliferation [15].

Keeping in mind the aforementioned data, our study concluded that both antihypertensive drugs, ACEI and ARB, are associated with an increased risk of lung cancer. In cases of continued usage, the doctors should do proper scanning of the lungs, via either x-ray or CT scan, at regular intervals to avoid any complications and late diagnosis. However, ideally, better alternatives should be suggested that do not have any major underlying side effects with longer use.

This study has several strengths. First, to our knowledge, with more than 20,000 patients followed for an average of seven years, this is the largest study to have been conducted to specifically assess this association in the local setting. Second, since it was a multi-center study, the sample was diverse. The study has several limitations as well. First, since it was a retrospective study, variables and information were limited. Second, even though smoking was considered, but information related to the number of packs per day was missing. Information on other confounding factors such as socio-economic status and occupational history were not available.

## Conclusions

In this study, we found an elevated risk of lung cancer with the long-term use of ACEIs and ARBs for hypertension. However, this association was relatively more among people using ACEIs than ARBs. Hence, patients undergoing long-term treatment with these anti-hypertensives need regular follow-up and proper scanning to avoid grave complications. Moreover, a high index of suspicion is required in patients developing respiratory symptoms or symptoms pertaining to lung cancer.
